# Middle-age-onset cerebellar ataxia caused by a homozygous *TWNK* variant: a case report

**DOI:** 10.1186/s12881-020-01002-4

**Published:** 2020-03-31

**Authors:** Kodai Kume, Hiroyuki Morino, Ryosuke Miyamoto, Yukiko Matsuda, Ryosuke Ohsawa, Yuhei Kanaya, Yui Tada, Takashi Kurashige, Hideshi Kawakami

**Affiliations:** 1 Department of Epidemiology, Research Institute for Radiation Biology and Medicine, Hiroshima University, 1-2-3 Kasumi, Minami-ku, Hiroshima, 734-8553 Japan; 2 Department of Clinical Neuroscience, Institute of Biomedical Sciences, Tokushima University Graduate School, 3-18-15 Kuramoto-cho, Tokushima, 770-0042 Japan; 3 Department of Neurology, National Hospital Organization Kure Medical Center and Chugoku Cancer Center, 3-1 Aoyama-cho, Kure, 737-0023 Japan

**Keywords:** *TWNK*, Cerebellar ataxia, Perrault syndrome

## Abstract

**Background:**

The *TWNK* gene encodes the twinkle protein, which is a mitochondrial helicase for DNA replication. The dominant *TWNK* variants cause progressive external ophthalmoplegia with mitochondrial DNA deletions, autosomal dominant 3, while the recessive variants cause mitochondrial DNA depletion syndrome 7 and Perrault syndrome 5. Perrault syndrome is characterized by sensorineural hearing loss in both males and females and gonadal dysfunction in females. Patients with Perrault syndrome may present early-onset cerebellar ataxia, whereas middle-age-onset cerebellar ataxia caused by *TWNK* variants is rare.

**Case presentation:**

A Japanese female born to consanguineous parents presented hearing loss at age 48, a staggering gait at age 53, and numbness in her distal extremities at age 57. Neurological examination revealed sensorineural hearing loss, cerebellar ataxia, decreased deep tendon reflexes, and sensory disturbance in the distal extremities. Laboratory tests showed no abnormal findings other than a moderate elevation of pyruvate concentration levels. Brain magnetic resonance imaging revealed mild cerebellar atrophy. Using exome sequencing, we identified a homozygous *TWNK* variant (NM_021830: c.1358G>A, p.R453Q).

**Conclusions:**

*TWNK* variants could cause middle-age-onset cerebellar ataxia. Screening for *TWNK* variants should be considered in cases of cerebellar ataxia associated with deafness and/or peripheral neuropathy, even if the onset is not early.

## Background

The Twinkle protein, encoded by the *TWNK* gene, is a mitochondrial helicase for DNA replication. The dominant *TWNK* variants cause progressive external ophthalmoplegia with mitochondrial DNA deletions, autosomal dominant 3 (PEOA3), while the recessive variants cause mitochondrial DNA depletion syndrome 7 (MTDPS7) and Perrault syndrome 5 (PRLTS5) [1]. PRLTS is characterized by sensorineural hearing loss in both males and females and gonadal dysfunction in females. Early-onset cerebellar ataxia and other neurological symptoms, including sensory neuropathy, muscle weakness, ophthalmoplegia, nystagmus, and intellectual disability, may appear in patients with PRLTS. Only two reports have described patients with PRLTS presenting with cerebellar ataxia at their middle ages [2, 3]. In MTDPS7, cerebellar ataxia is characterized by infantile onset [4]. PEOA3 rarely causes cerebellar ataxia [5]. We report a homozygous *TWNK* variant in a patient with middle-age-onset cerebellar ataxia associated with deafness and sensory neuropathy.

## Case presentation

**Fig. 1:**
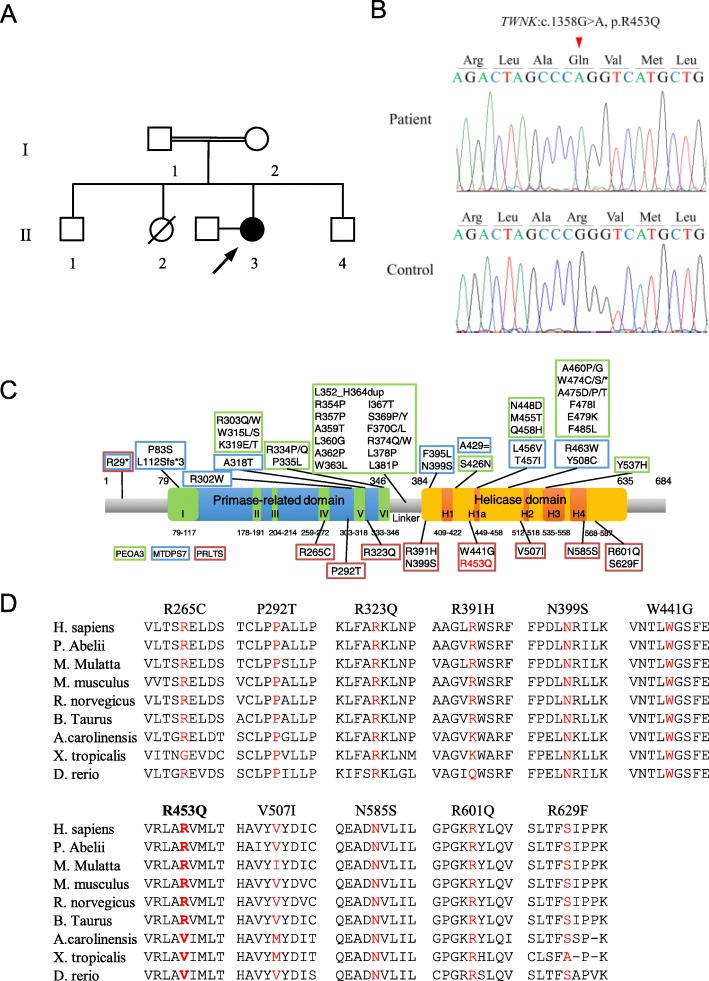
Identification of the *TWNK* variant. **a** Family tree in this study. The filled circles indicate affected individuals, and the open circles and boxes indicate non-affected individuals. The proband is indicated by an arrow. **b** Sanger sequences of the *TWNK* variant in the patient and a control subject. **c** Domain architecture of Twinkle and previously reported variants for three phenotypes: dominant progressive external ophthalmoplegia type 3 (PEOA3); recessive mitochondrial DNA depletion syndrome 7 (MTDPS7), and Perrault syndrome 5 (PRLTS5). The variant in our case is marked in red. **c** Conservation of protein sequence at the nine residues with variants. Mutated residues are marked in red. The variant in our case is marked in bold

A Japanese female presented hearing loss at age 48, a staggering gait at age 53, and numbness in her distal extremities at age 57. Her parents were consanguineous, and she had three siblings without neurological symptoms and no children (Fig. 1a). Neurological examination at age 58 revealed gaze-directional nystagmus, ataxic dysarthria, severe sensorineural hearing loss, decreased deep tendon reflexes, cerebellar incoordination in the limbs, sensory disturbance in the distal extremities, positive Romberg’s sign, and inability to walk in tandem. Laboratory tests at age 58 showed no abnormal findings, including in CK, lactic acid, and pyruvate levels. A moderate elevation of pyruvate concentration levels was observed at age 69 (0.98 mg/dL, normal range: 0.30–0.94 mg/dL). Magnetic resonance imaging showed mild cerebellar atrophy. We performed exome sequencing using SureSelect Human All Exon V6 and a HiSeq 2500 sequencer. We used BWA (http://bio-bwa.sourceforge.net) for mapping, SAMtools (http://samtools.sourceforge.net) and Picard (http://www.htslib.org) for SAM/BAM handling, GATK (https://gatk.broadinstitute.org), SAMtools, and Pindel (http://gmt.genome.wustl.edu/packages/pindel/) for variant calling, and ANNOVAR (http://annovar.openbioinformatics.org) for variant annotation. We detected 109,876 variants and used filtering criteria consisting of zygosity, function, allele frequencies in open databases, regions of runs of homozygosity, and Combined Annotation Dependent Depletion (CADD) score (http://cadd.gs.washington.edu/home). After filtering, we identified two homozygous variants: *TWNK* (NM_021830):c.1358G>A,p.R453Q (rs760988188); and *TMBIM4* (NM_016056):c.713delA,p.K238Sfs*3 (rs767782535). We determined that the *TWNK* variant was causative, as it has been reported that *TWNK* variants cause cerebellar ataxia, sensorineural deafness, and sensory neuropathy, which our patient presented. We validated this variant using Sanger sequencing (Fig. 1b). A segregation study was not conducted because we were unable to contact family members. The heterozygous variant has been reported as a very rare variant in gnomAD (https://gnomad.broadinstitute.org/; allele frequency in East Asian: 0, total: 0.000003976), whereas the homozygous variant was not found in the database. The variant pathogenicity was confirmed by three prediction tools: CADD score, 23.4; PolyPhen-2 (http://genetics.bwh.harvard.edu/pph2/), possibly damaging; and MutationTaster (http://www.mutationtaster.org/), disease causing. The variant was classified as likely pathogenic according to the guidelines of the American College of Medical Genetics and Genomic and the Association for Molecular Pathology [6].

## Discussion and conclusions

We reported a case of middle-age-onset cerebellar ataxia caused by a homozygous *TWNK* variant. Because we could not confirm gonadal dysfunction, we were not able to diagnose PRLTS. However, cerebellar ataxia associated with hearing loss and sensory neuropathy in our patient was consistent with the neurological symptoms of PRLTS. PRLTS is classified as PRLTS1 to PRLTS6, caused by genes *HSD17B4*, *HARS2*, *CLPP*, *LARS2*, *TWNK*, and *ERAL1* [1, 7]. We first reported that *TWNK* was a causative gene for PRLTS5 in two families of Japanese and European ancestry [1]. According to our original report and subsequent studies [3, 8–12], cerebellar ataxia in patients with PRLTS5 starts between ages 3 and 43, and hearing loss develops between ages 3 and 13 (Table 1). In contrast, our patient presented cerebellar ataxia at age 53 and hearing loss at age 48. Of the previously reported *TWNK* variants, eight are located in the helicase domain and three in the primase-related domain (Fig. 1c). The three families with the primase-related domain variants (families III, IV, and VII) tended to have a younger age onset than those with the helicase domain variants (Table 1). On the other hand, among the six families with helicase domain variants, families other than family VI and our patients had at least one variant located at a fully conserved amino acid in vertebrates (Fig. 1d). Patients in family VI presented cerebellar ataxia at older ages, although they had a nonsense variant in another allele (Table 1). Because the mutated amino acid in family VI and in our patient is less conserved in vertebrates, dysfunction of helicase activity may not be as severe as in other families. Therefore, our patient may have developed at an older age due to a less damaging variant located in the helicase domain of Twinkle.

**Table 1:** Genetic and clinical features in PRLTS5 cases

Family	Sex	Variant	CADD	Gonadal dysfunction	Hearing loss	Cerebellar ataxia	Neuropathy	Epilepsy	Reference
I	F	p.R391H, **p.N585S**	22.8, 24	+	+ (13)	+ (20)	N.A.	N.A.	1
	F	p.R391H, **p.N585S**	22.8, 24	+	+ (8)	+ (16)	N.A.	N.A.	1
II	F	**p.W441G**, p.V507I	29.7, 18.57	+	+ (7)	+ (teens)	+ (20)	+ (7)	1
	F	**p.W441G**, p.V507I	29.7, 18.57	+	+ (7)	+ (N.A.)	+ (N.A.)	N.A.	1
III	F	**p.R323Q**, **p.N399S**	32, 17.59	+	+ (3)	+ (N.A.)	+ (N.A.)	N.A.	8
IV	F	p.R265C, p.R265C	24.3, 24.3	+	+ (3)	+ (N.A.)	+ (N.A.)	N.A.	9
	F	p.R265C, p.R265C	24.3, 24.3	+	+ (3)	+ (N.A.)	+ (N.A.)	N.A.	9
	M	p.R265C, p.R265C	24.3, 24.3	N.A.	+ (3)	+ (N.A.)	+ (N.A.)	N.A.	9
V	F	**p.N399S**, **p.R601Q**	32, 25.3	+	+ (5)	+ (3)	+ (N.A.)	–	10
	F	**p.N399S**, **p.R601Q**	32, 25.3	+	+ (12)	+ (11)	+ (N.A.)	–	10
VI	F	p.R29*, p.S629F	32, 25.3	+	+ (5)	+ (35)	+ (N.A.)	N.A.	3
	F	p.R29*, p.S629F	32, 25.3	+	+ (3)	+ (43)	+ (N.A.)	N.A.	3
	M	p.R29*, p.S629F	32, 25.3	N.A.	+ (4)	+ (20)	+ (N.A.)	N.A.	3
VII	M	**p.P292T**, **p.P292T**	22.1, 22.1	N.A.	+ (4)	+ (8)	+ (N.A.)	–	11
VIII	F	**p.N399S**, p.R453Q	32, 23.4	+	+ (4)	+ (12)	+ (N.A.)	N.A.	12
IX	F	p.R453Q, p.R453Q	23.4, 23.4	N.A.	+ (48)	+ (53)	+ (57)	N.A.	Our case

The variants located in fully conserved amino acids in vertebrates are marked in bold. The numbers in parentheses indicate ages of onset

*Abbreviations: CADD* Combined Annotation Dependent Depletion, *N.A.* not available

R453, where the variant in our patient is located, may be important for Twinkle functions. It lies in conserved helicase motif H1a, which is involved in ATP binding and hydrolysis. In addition, it interacts with L381, where the variant causing PEOA3 is located. Functional analysis revealed that the L381P variant reduces the ATPase and helicase activities of Twinkle [13]. Collectively, the R453Q variant may lead to Twinkle ATPase and helicase dysfunction.

In conclusion, TWNK variants may cause middle-age-onset cerebellar ataxia. Therefore, screening for TWNK variants should be considered in cases of cerebellar ataxia with hearing loss and/or sensory neuropathy, even if the onset is not early.

## Data Availability

The datasets used and/or analyzed during the current study are available from the corresponding author on reasonable request. The sequence datasets generated during the current study are not publicly available because it is possible that individual privacy could be compromised.

## Abbreviations

CADD: Combined Annotation Dependent Depletion
MTDPS7: Mitochondrial DNA depletion syndrome 7
PEOA3: Progressive external ophthalmoplegia with mitochondrial DNA deletions, autosomal dominant 3
PRLTS: Perrault syndrome
